# Implementation and Evaluation of Life-planning Lecture to Enhance Perspective-Taking among High School Students: A School-based Nonrandomized Waitlist Intervention Study in Japan

**DOI:** 10.31662/jmaj.2021-0033

**Published:** 2021-10-01

**Authors:** Kazuyo Watanabe, Aya Goto, Kayoko Ishii, Hiromi Yoshida-Komiya, Shinya Ito, Misao Ota

**Affiliations:** 1 International Community Health, Fukushima Medical University Graduate School of Medicine, Fukushima, Japan; 2 Preparing Section for School of Midwifery, Fukushima Medical University, Fukushima, Japan; 3 Center for Integrated Science and Humanities, Fukushima Medical University, Fukushima, Japan; 4 Department of Midwifery and Maternal Nursing, Fukushima Medical University School of Nursing, Fukushima, Japan; 5 Center for Gender Specific Medicine, Fukushima Medical University, Fukushima, Japan; 6 Kitasato University School of Nursing, Kanagawa, Japan

**Keywords:** Empathy, School health, Adolescent, Reproductive health

## Abstract

**Introduction:**

The Australian “empathy session,” which is a parenting program aimed at alleviating postpartum depression by increasing empathy among expecting couples, was adapted to a life-planning education program for Japanese high school students. In this present study, we aimed to assess changes in high school students’ empathy levels.

**Methods:**

A nonrandomized, controlled, waitlist intervention was performed in 210 first-year students. They were divided into intervention and waitlist control groups. The life-planning lecture consisted of two parts: (1) reproductive health and (2) empathy and communication skills. The main outcome indicator was the Perspective-Taking subscale of an empathy index. Logistic regression was used to examine the association between the intervention and change in the Perspective-Taking scale score controlling for background factors.

**Results:**

As per our findings, a significant difference was noted in the scale scores of Perspective-Taking before and after the program within the intervention group (3.76 ± 0.61 before the lecture and 3.86 ± 0.64 after the lecture; *P* = 0.01). In the between-group analysis, the likelihood of an increase in the scale score of Perspective-Taking was significantly higher in the intervention group (OR = 2.29, 95 % confidence interval = 1.23-4.26).

**Conclusions:**

Japanese high school students’ Perspective-Taking improved through learning reproductive life-planning and communication skills.

## Introduction

The World Health Organization (WHO) has recommends ten skills (including empathy) as important life skills that should be included in education in combination with health information ^(1)^. Life skills, as defined by the WHO, are abilities for adaptive and positive behaviors that enable individuals to deal with the demands and challenges of everyday life effectively ^(1)^. In particular, life skills are psychosocial competencies and interpersonal skills that help people make informed decisions, solve problems, think critically and creatively, communicate effectively, build healthy relationships, empathize with others, and cope with managing their lives in a healthy and productive manner. Life skills may be directed toward personal actions, actions toward others, or actions that alter the surrounding environment to make it conducive to health ^(2)^. Empathy building is one’s ability to listen, understand another’s needs and circumstances, and express that understanding. It can help us accept others who may be very different from ourselves and thus improves social interactions and motivates aiding and prosocial behaviors and suppresses antisocial behavior ^(2)^. Along the same lines with the WHO’s life skills promotion, the World Medical Association’s Declaration of Ottawa on child health recommends that a healthy, safe, and sustainable not only physical, but also emotional environment is necessary for a child’s development.

Adolescents represent a significant and unique stage of development in that they are still looked after by others as they simultaneously prepare to look after the next generation. The most significant mental health challenge among adolescents in Japan is teen suicide; in fact, 659 cases were reported in 2019 ^(3)^. The causes of teen suicide include problems at school, bullying, and health problems ^(4)^. While adolescence is a period in which individuals develop an interest in themselves and others as the mind and body develop, it is also a time when interpersonal problems can arise because of poor decision-making and lacking communication skills. Adolescents can also be susceptible to depression, which can have long-term impacts on an individual’s health and life course ^(5)^. Therefore, it is important to implement life skills education to improve social interaction during this time.

Another challenge in reproductive health is teen pregnancy. In Japan, abortions peaked around 2001 and have been decreasing since; however, the percentage of teens that carry a pregnancy to birth has seen a slight increase^(4), (6)^. According to a report on child abuse, 27 % of child deaths immediately after birth are perpetrated by mothers aged 19 years or younger ^(7)^. In a previous study, it was shown that family planning is important to prevent child abuse ^(8)^. Since pregnancy and parenting are continuous, it is important to plan pregnancy. A Japanese national campaign for the health of mothers and children, “Healthy Parents and Children 21 (Second Phase),” prioritizes preparing adolescents for adulthood and providing continuous support during the perinatal period. It is essential to promote decision-making among adolescents with regard to pregnancy and parenting as they foster the next generation. Furthermore, improving an individual’s self-awareness of the importance of life, as well as their level of understanding and acceptance of others, can be effective in improving interpersonal relationships.

As per the Cochrane systematic review in 2016, evidence that sex education interventions for adolescents reduce sexually transmitted infections and pregnancy was lacking ^(9)^. An Australian study reported a program (referred to as “empathy sessions” below) that encouraged husbands to have a better understanding and more empathy during pregnancy, which was found to suppress postpartum depression in mothers ^(10)^. We have prepared a Japanese protocol for this empathy session and are implementing it widely ^(11)^. The empathy session targets couples who are expecting a child; it contains exercises wherein the participants can share their current problems and improve their prospects for life following birth ^(12)^. The original session was developed for expecting couples in Australia ^(10)^ and adapted to a Japanese public health service setting. The tools were modified, taking into account cultural and social factors (e.g., the partner’s time of returning home from work in the discussion scenario was revised from 6 p.m. in the original to 8 p.m. in the Japanese version), to fit with Japanese parenting and family styles ^(11), (12)^.

Subsequently, a small trial that used the empathy session as a role-play for being parents among university students was implemented, and an increase in their empathy levels was observed ^(13)^. However, the empathy session has not yet been tested in younger adolescents. Previous studies have shown that adolescents, especially girls, are at a high risk of depression, which is often associated with negative life events, life stress, and low self-esteem ^(14), (15), (16)^. The purpose of the empathy session was to increase mutual understanding and identify behaviors that would help each other in times of stress ^(10)^. The session was reported to be effective in reducing depression, especially for women with low self-esteem, and we thought it would address the learning needs of adolescents. Thus, in this present study, a life-planning lecture was prepared for first-year high school students by incorporating the empathy session exercises into the existing reproductive health class at the school. The intervention aimed to increase empathy in order to promote mutual understanding among the students and to facilitate life planning. This study, applying a non-randomized, waiting list intervention design, aimed to assess whether a life-planning lecture incorporating the “empathy session” could improve Japanese high school students’ empathy.

## Materials and Methods

### Participants

The research design was a school-based, nonrandomized, waitlist intervention study. All students (N = 210) in first year (grade 10) at High School A in Fukushima Prefecture were targeted. High School A was a coeducational school where the lead author gave a reproductive health lecture to the first-year high school students every year. The participants were divided into two groups: the intervention group and the waitlist control group. Grouping was decided after a pilot trial and by consulting a school nurse in charge of health education. Prior to the intervention, program staff conducted two pilot trials that are explained below ^(17)^. The size of the two groups was then made comparable. The intervention group consisted of 109 students who provided informed consent, whereas the waitlist control group consisted of 101 students who also provided informed consent. The exclusion criterion was data with missing values for Perspective-Taking, which was the main indicator in this study. Therefore, 103 participants in the intervention group and 96 in the waitlist control group who provided informed consent were included in the analysis.

### Procedure

This was an interventional study, and data for the intervention group and waitlist control group before and after the intervention were compared. Students in the waitlist control group also received the same intervention after the study was completed. The school’s intention was that all students could experience this new trial by treating the control group as a waitlist group for the intervention. The data for this study were collected on March 14, 2019. A 90-min life-planning lecture was conducted with the intervention group and the waitlist control group on the same day at different times. The intervention group received the life-planning lecture first. The classroom (gym) was the same for both groups, and the groups of students were switched during the break. An important point considered in the implementation of this intervention was to eliminate contamination between the intervention and waitlist control groups. Therefore, we have designated a separate entrance and exit for the venue to make sure students did not exchange information when the two groups were being switched.

The developer of the original intervention added the empathy session to a regular parenting class for couples, whereas this present study incorporated the empathy session into the existing standard reproductive health session at a high school. The life-planning lecture had two parts. The first half was the routine didactic teaching on reproductive health, whereas the latter half was the empathy session. The empathy session included discussion exercises about parenting with students role-playing as couples (Table 1). These core activities were kept the same as in the original program ^(10)^. Minor adaptations for high school students included shortening the time allocated for group discussion to accommodate a lecture to explain about pregnancy and role-playing as couples when discussing. Prior to the intervention, program staff conducted two pilot trials at a university ^(17)^ and a target high school in order to confirm acceptance of the program content by students and high school teachers. With regard to logistics, the appropriate class size, readability of the evaluation questionnaire, class flow, timelines, and ways to avoid contamination between two groups were assessed.

**Table 1.** Structure of the Life-planning Lecture.

Objectives and Content	
Reproductive session (42 min)	Didactic teaching
	1. Introduction
	2. Importance of life
	3. Own growth and interpersonal relationship
Break (8 min)	
Empathy session (30 min) (Preparing for parenting) • Agreement and disagreement with others • Respecting opinions • Discuss parenting in a mock couple	Work 1: Confirm one’s role • Discuss an assigned role (mother or father) (2 min)
	Work 2: Discuss the concern checklist of expected parenting problems • Fill out the checklist individually (2 min) • Discuss in a mock couple (5 min) • Share opinions to the whole class (5 min) • Present the top five concerns (1 min)
	Work 3: Discuss the scenario of “the difficult day” • Confirm the setup (1 min) • Discuss solutions with several mock couples (5 min) • Share opinions with the whole class (5 min) • Explanation of confusions and solutions for first-time parents (2 min)
Summary (10 min)	The second questionnaire
	Lecture evaluation questionnaire

The assessment method was as follows. The questionnaires were administered to each group immediately before and after the intervention (or a regular class). The waitlist control group had a different class, while the intervention group was taking the life-planning lecture. The waitlist control group took the first questionnaire before this different class, the second questionnaire was conducted at the beginning of the life-planning lecture, and the third questionnaire was conducted at the end of the life-planning lecture. Classes at the school lasted for 90 min. There were two facilitators in both classes: one midwife (the lead author) and the school nurse. A trial lecture was performed among the facilitators prior to the intervention.

### Instruments

A questionnaire that included the following seven components was prepared: individual attributes, empathy, mental health, pregnancy planning, satisfaction with school life, subjective health, and evaluation of the lecture. Individual attributes included sex, hometown (Fukushima Prefecture or not), and whether they were living with someone (with family, alone, or in a dormitory).

As the index of mental health to be considered as one of the important confounding factors, the Face Scale was utilized. The Face Scale is described as an index with verified reliability and validity developed by Lorish and Maisiak as a method to evaluate temporal mood. The Face Scale has a very happy face at No. 1 to a very sad face at No. 20, wherein a respondent chooses one face that most expresses their current emotional state ^(18)^.

As another significant confounding factor, participants were asked about the extent to which they were satisfied with their school life, by using an item from “The Second Basic Survey on Life and Awareness of Youth” by the Cabinet Office ^(19)^. The answer option was a 4-point scale from 1 (satisfied) to 4 (not satisfied). “Satisfied” was considered to comprise responses of 1 and 2, whereas “unsatisfied” comprised 3 and 4. The subjective health question was extracted from a prefecture-wide health survey conducted by Fukushima Prefecture ^(20)^. Whether a student thinks oneself healthy or not was asked, and the answer option was a 5-point scale from 1 (I agree strongly) to 5 (I do not agree at all). “Healthy” was considered to include responses 1 and 2, whereas responses 3, 4, and 5 indicated “unhealthy.”

As for the main outcome indicator to assess the empathy level, the “Perspective-Taking” subscale of the Multidimensional Empathy Scale (MES) developed and validated by Suzuki and Kino was used ^(21)^. The MES is a scale that evaluates empathy through a multidimensional structure that focuses on the discrimination of self- and other-oriented nature in the cognitive and emotional dimensions. The MES has 24 questions as self-report measures, consisting of 5 subscales―“Other-Oriented Emotional Reactivity,” “Self-Oriented Emotional Reactivity,” “Emotional Susceptibility,” “Perspective-Taking,” and “Fantasy.” Each subscale can be used independently ^(22), (23)^. In this present study, the Perspective-Taking scale, which indicates one’s understanding of others as the subject of observation, by placing oneself in their position, was used. In other words, it indicates an ability to accept others’ positions by suppressing ideas centered on oneself, which was consistent with the expected effect of the empathy session. In addition, minimizing the number of questions was also of advantage in reducing the burden placed on participants and thereby increasing the practicability of the intervention. The scores of the Perspective-Taking scale were from 1 to 5 (5-point scale: 1, Not true at all; 2, Not really true; 3, Neither; 4, Somewhat true; and 5, Very true). Scores of the reverse items were reversed when scoring, and a higher calculated total score indicated a higher empathy level. The mean score of the Perspective-Taking scale described by Suzuki and Kino was 3.47 (SD = 0.62), with no sex difference ^(21)^. In our previous study conducted among university students, the mean score of the Perspective-Taking scale was 3.84 (SD = 0.46) ^(17)^. Cronbach’s alpha for this measurement in the present participants was 0.81.

The participants were then asked to evaluate the lecture at the end. The evaluation included three questions about class management (about the materials distributed, allocated time, and facilitation), with three other questions on content (“It gave me an opportunity to value my present life,” “It made me think about the future,” and “It would be useful for having a good relationship”). Students were also asked to answer using a 5-point scale (“Very true,” “True,” “Neither,” “Not true,” and “Not true at all”). In addition, there was a comment section where participants could describe their opinions on the life-planning lecture.

### Data analysis

The main outcome indicator was the Perspective-Taking score. First, individual attributes, mental health, satisfaction with school life, and subjective health were compared between the two groups (intervention and waitlist control) using the chi-squared test. For the pre-intervention values for Perspective-Taking, the data from the first questionnaire from the intervention and control groups were used, and the scores were compared using the Mann-Whitney *U* test. For comparing Perspective-Taking before and after the intervention, the data from the first and second questionnaires for each group were analyzed using the Wilcoxon signed-rank test. Since the total score of Perspective-Taking did not follow a normal distribution, the changes in its values were classified as “increased” and “unchanged/decreased,” and whether there was a significant difference between the control and intervention groups was also analyzed, controlling for background factors that were significant on univariate analysis. Multivariate analysis was performed with binominal logistic regression by entering the dichotomized value of the change in Perspective-Taking (increased (1) and unchanged/decreased (0)) as a dependent variable. The effect size of each factor entered into the multivariate analysis was assessed by calculating Cohen’s r. For the statistical analysis, IBM SPSS Statistics version 25 was used, with a significance level of 5 %.

### Ethical considerations

This present study was approved by the ethics committee of Fukushima Medical University (No. 30050). A printed explanation sheet was distributed to students and their guardians, and it was explained face-to-face to students before the lecture. The anonymous questionnaire in a sealed envelope was posted into a collection box. Returning a completed questionnaire was taken as providing informed consent to participate in the study.

## Results

The characteristics of participants for the first questionnaire are shown in Table 2. There were significant differences between the intervention group and the control group in sex (*P* = 0.002) and mental health (*P* = 0.04). Regarding sex, there were more male subjects in the intervention group than in the control group. Mental health was also examined by dividing subjects into groups with a score of 1-10 and 11-20. The intervention group had more subjects with better mental health than the control group. No differences were noted between the groups in participants’ hometown, the people they live with, satisfaction with school life, and subjective health.

**Table 2.** Characteristics of Subjects: Intergroup Comparison.

		N = 199	n (%)^a^		
			Control group (n = 96)	Intervention group (n = 103)	*P*-value^b^
Sex	Female	146	79 (84.0)	67 (65.0)	0.002
	Male	51	15 (16.0)	36 (35.0)
Hometown	Fukushima Prefecture	182	88 (92.6)	94 (91.3)	0.72
	Outside of Fukushima Prefecture	16	7 (7.4)	9 (8.7)
Living with	Parents	191	93 (97.9)	98 (95.1)	0.45
	Alone/dormitory	7	2 (2.1)	5 (4.9)
Mental health^c^	1-10	169	77 (81.1)	92 (91.1)	0.04
	11-20	27	18 (18.9)	9 (8.9)
Satisfaction with school life^d^	Satisfied (1, 2)	150	76 (80.0)	74 (72.5)	0.22
	Not satisfied (3, 4)	47	19 (20.0)	28 (27.5)
Subjective health^e^	Healthy (1, 2)	120	52 (54.7)	68 (66.0)	0.10
	Not healthy (3-5)	78	43 (45.3)	35 (34.0)

^a^ 100 % does not mean the total of all subjects as there were missing values. ^b^ The chi-squared test or Fisher’s exact test. ^c^ An emotion evaluation method with the scale ranging from 1 to 20. A higher number means a sadder face. ^d^ 4-point scale: (1) Very satisfied, (2) Satisfied, (3) Not very satisfied, (4) Not satisfied. ^e^ 5-point scale: (1) Very true, (2) True, (3) Neither, (4) Not true, (5) Not true at all.

Intergroup comparison of the Perspective-Taking scale score showed that there was no difference in these scores before the intervention (Table 3). An intragroup comparison of the Perspective-Taking scale score showed that there was a significant difference in the intervention group between before (3.76 ± 0.61) and after (3.86 ± 0.64) the intervention (*P* = 0.01) (Table 3). There was no significant difference in scale scores in the control group.

**Table 3.** Comparison of the Perspective-Taking Scale Score before and after the Intervention: Intragroup Comparison and Intergroup Comparison.

	Control group^a^ (n = 96)			Intervention group^a^ (n = 103)			*Intergroup baseline comparison* *P*-value^c^
	Before lecture (1st)	Before lecture (2nd)	*Intragroup comparison* *P*-value^b^	Before lecture	Immediately after lecture	*Intragroup comparison* *P*-value^b^	
Scale score	3.8 (1.6-5.0)	3.8 (1.2-5.0)	0.43	3.8 (1.6-5.0)	3.8 (1.8-5.0)	0.01	0.73
	3.78 ± 0.70	3.76 ± 0.76		3.76 ± 0.61	3.86 ± 0.64		

^a^ Median (range). The mean and standard deviations are also shown for the scale score. ^b^ Wilcoxon signed-rank test. ^c^ Mann-Whitney U test.

Confounding factors, sex and mental health, were force entered in a binomial logistic regression analysis, and the probability of an increased Perspective-Taking scale score increased significantly for the intervention group, with an odds ratio of 2.29 (95 % confidence interval = 1.23-4.26) (Table 4). Cohen’s r for each categorical item was 0.18 for the intervention condition (0.02 for sex and 0.06 for mental health), indicating a small effect size.

**Table 4.** Relationship between the Difference in the Perspective-Taking Scale Score before and after the Intervention and Participants’ Attributes.

	Post value minus pre-value n (%)		Univariate^a^	Multivariate^b^		
Item	Increase	Unchanged/Decrease	*P*-value	OR	95 % CI	*P-*value
By group						
Control group	26 (35.1)	70 (56.0)	0.004	1.00		
Intervention group	48 (64.9)	55 (44.0)		2.29	1.23-4.26	0.01
Sex						
Female	56 (75.7)	90 (73.2)	0.70	1.00		
Male	18 (24.3)	33 (26.8)		1.49	0.74-3.02	0.27
Mental health						
1-10	64 (88.9)	105 (84.7)	0.41	1.00		
11-20	8 (11.1)	19 (15.3)		1.22	0.49-3.05	0.67

^a^ Chi-squared test. ^b^ Binominal logistic regression analysis. Dependent variable: increase = 1, unchanged/decrease = 0.

In the evaluation of participants’ satisfaction with the life-planning lecture, the cumulative evaluation of the six questions on the distributed documents (96 %), lecture progress (93 %), and contents (90 %) were mostly “Very true” and “True.”

## Discussion

Previous studies in Australia and Japan found that empathy sessions were effective in preventing postpartum depression in Australian and Japanese couples and in improving the empathy level of Japanese university students. This current study is an addition to the existing evidence showing that the session had a short-term positive effect in improving empathy as measured by the Perspective-Taking scale in Japanese high school students. The present school health trial was characterized by the use of a waitlist control group, in contrast to much health education research in Japan, which often does not have a control group. This study design was made possible by the enthusiasm of the school teachers to scientifically determine the effects of this intervention based a long-term collaboration with a local university. In terms of didactic method, interactive activities were appealing to both teachers and students. More importantly, the intervention content was relevant to the issues that the school faced, including bullying and pregnancy.

The life-planning lecture was well-accepted by first-year high school students. Activities of the empathy session were also well received by the postpartum depression prevention class for expectant couples, and opinions were exchanged actively therein ^(11)^. The main reason for the high level of acceptance by students was that the lecture content was well suited to the objective of showing the importance of life, self-growth, interpersonal relationships, and preparation to become a parent (Table 1). This was consistent with the needs of young participants who are forming their identities ^(24)^. The second reason was that the lecture approach addressed the real-life application of essential knowledge, attitudes, and skills and used interactive teaching and learning methods. Studies suggest that adolescents often regard peers as an important source of information they need ^(25)^.

There was a significant increase in scores on the Perspective-Taking scale after the intervention. When a small number of male students were removed from the intervention group, and analysis included only female students in the intervention group, there was still a significant difference (*P* = 0.02). After adjusting for sex and mental health, the intervention group had a 2.3 times higher Perspective-Taking increase compared with the control group. In a previous study of pregnant couples, no significant difference was observed in the Perspective-Taking scale score before and after the Japanese empathy session (mothers: *P* = 0.70, fathers: *P* = 0.60) ^(12)^. Although the evaluation index was different in another previous study, which was a parenting preparation study for university students, an improvement in empathy was observed, as in the present study ^(13)^. During the first year of high school when students are around 15 years old, they are developing social Perspective-Taking, and they are able to learn roles through interactions ^(26)^. In the empathy session, students in a group discussed their own opinions and compared them with those of their peers for two assigned parenting exercises. According to the theory of communication, peer education has the advantage of strong cultural suitability and universal acceptability ^(27)^. The empathy session appears to have had an impact on students’ understanding of others by putting oneself in another’s position. In addition, since increased empathy has been associated with increased prosocial behavior and decreased aggression ^(28)^, an intervention to increase empathy has potential to contribute to the prevention of bullying and suicide. Concerning this present study, however, empathic concern rather than the perspective-taking component in the overall empathy scale is more related to the deterrence of aggression ^(29)^, and we plan to include this subscale in our further research.

### Limitations

This present study was limited in so far as only students of one high school in an urban area in a northern prefecture were tested, and the effect size of intervention status was small. For further research, efforts are needed to increase the number of high schools in diverse locations with a better sex balance. We further plan to attempt to expand the project at the community level in collaboration with a local government and a board of education ^(30)^. In addition, which component of the lecture worked to increase the empathy of students could not be specifically evaluated. Thus, more rigorous study is needed, for example, to compare lectures with and without the empathy session to clarify the mechanism.

### Conclusions

In this present study, we have set up a control group from among all first-year students at a high school and conducted a nonrandomized, controlled waitlist intervention. The result showed that Perspective-Taking improved following a life-planning lecture that combined reproductive health and an empathy session. Improvement in Perspective-Taking entails an improvement in understanding others, whereby empathetic interest increases, and social interaction with others becomes smoother. This life-planning lecture may help educate students in reproductive health and prepare them as members of society who can engage well in parenting.

## Article Information

### Conflicts of Interest

None

### Sources of Funding

This work was supported by Fukushima Medical University (FMU) and FMU School of Nursing, and JSPS KAKENHI grant number JP20K03212.

### Acknowledgement

The authors would like to thank the teachers who supported the planning and evaluation of this trial. The authors would also like to thank the reviewers for their insightful comments. This work was supported by research grants from Fukushima Medical University (FMU) and FMU School of Nursing, and JSPS KAKENHI Grant Number JP20K03212.

### Author Contributions

Kazuyo Watanabe: Conceptualization, data collection, analysis, funding acquisition, writing, editing

Aya Goto: Conceptualization, data curation, project administration, supervision, review and editing

Kayoko Ishii: Results interpretation, review and editing

Hiromi Yoshida-Komiya: Project administration, results interpretation, review and editing

Shinya Ito: Analysis, results interpretation, review and editing

Misao Ota: Results interpretation, supervision, review and editing

### Approval by Institutional Review Board (IRB)

IRB approval cord and name of the institution

The ethics committee of Fukushima Medical University (No. 30050)
